# Infected pericardial effusion caused by *Prevotella intermedia*: a rare diagnosis

**DOI:** 10.3389/fcimb.2025.1612282

**Published:** 2025-06-16

**Authors:** Wan Xu, Dan Han, Tianyang Chen, Qian Wang

**Affiliations:** ^1^ Songnan Community Health Service Center, Shanghai, China; ^2^ Department of Emergency Internal Medicine, Shuguang Hospital Affiliated to Shanghai University of Traditional Chinese Medicine, Shanghai, China

**Keywords:** *Prevotella intermedia*, pericardial tamponade, pericardial effusion, infection, mNGs

## Abstract

*Prevotella intermedia* is a Gram-negative bacterium that thrives in anaerobic environments. It presents challenges in detection through routine laboratory assays, and hitherto, there has been no documented instance of detecting this bacterium in pericardial effusion in China. Metagenomic Next-Generation Sequencing (mNGS) can boost the detection rate of this pathogen and furnish early guidance for clinical management.

## 1 Case summary

A 70-year-old male with a past medical history of diabetic nephropathy, stage 5 chronic kidney disease (CKD stage 5), heart failure, and hypertension was admitted. The patient presented to the clinic with fever and dyspnea. A chest computed tomography (CT) scan demonstrated massive pericardial effusion, leading to the diagnosis of pericardial tamponade. Subsequently, pericardiocentesis and drainage were performed. Microbiological examination of the drained fluid specimen identified *Prevotella intermedia* infection. Following anti-infective treatment, the patient’s condition ameliorated, and he was discharged. This represents the first reported case of pericardial effusion due to *Prevotella intermedia* infection in China. It is postulated that the infection might have stemmed from the oral cavity or a hemodialysis catheter.

## 2 Conclusion

The latent peril of *Prevotella intermedia* as an opportunistic pathogen in patients with underlying comorbidities should not be overlooked. This case accentuates the diagnostic complexity in discriminating between infected and non-infected pericardial effusions and highlights the pivotal role of an etiological basis in clinical decision-making.

## 3 Synopsis

Pericardial disorders commonly present as acute pericarditis, pericardial effusion, and constrictive pericarditis. In cases where pericardial tamponade occurs as a complication, prompt intervention is of utmost importance to mitigate mortality. Pericardial effusions can be categorized as either infectious or non-infectious, depending on their etiologies. In developed nations, infectious pericardial effusions, predominantly caused by gram-positive microorganisms, constitute the leading cause of acute pericarditis. In developing countries, given the high prevalence of HIV, tuberculosis emerges as a prevalent causative factor (Anna et al., 2021).The genus Prevotella encompasses a variety of Gram-negative anaerobic bacteria that are widely distributed throughout the human body. Different species of Prevotella have been detected and isolated from the human oral cavity, respiratory tract, vagina, and other sites. Notably, the oral cavity harbors the greatest number of known Prevotella species in humans. Currently, no species within this genus is recognized as being solely pathogenic; however, its members have been implicated in numerous diseases (Jiyeon et al., 2016; Tett et al., 2021). *Prevotella intermedia* frequently functions as an opportunistic pathogen in the respiratory and oral cavities (Teanpaisan et al., 1996; Vijayalakshmi Sharadindu et al., 2025). The sampling of *Prevotella intermedia* is subject to multiple influencing factors, including sampling time, exposure of specimens to air, and transportation conditions, which collectively contribute to a low positive detection rate in routine cultures. In China and Korea, hitherto, only cases of intracranial infection attributed to *Prevotella intermedia* have been reported. This article represents the first account in China of a case involving pericardial tamponade resulting from *Prevotella intermedia* infection.

## 4 Case presentation

A 70-year-old male was admitted to the hospital with the chief complaint of “fever accompanied by shortness of breath for one week”. This symptom served as the immediate trigger for his hospitalization and paved the way for the ensuing diagnostic and treatment procedures. The patient had a pre-existing medical history of diabetic nephropathy, CKD stage 5, heart failure, and hypertension. He had been undergoing regular hemodialysis treatment in the nephrology department for an extended period, which constituted an important backdrop to his current state.

**Figure 1:**
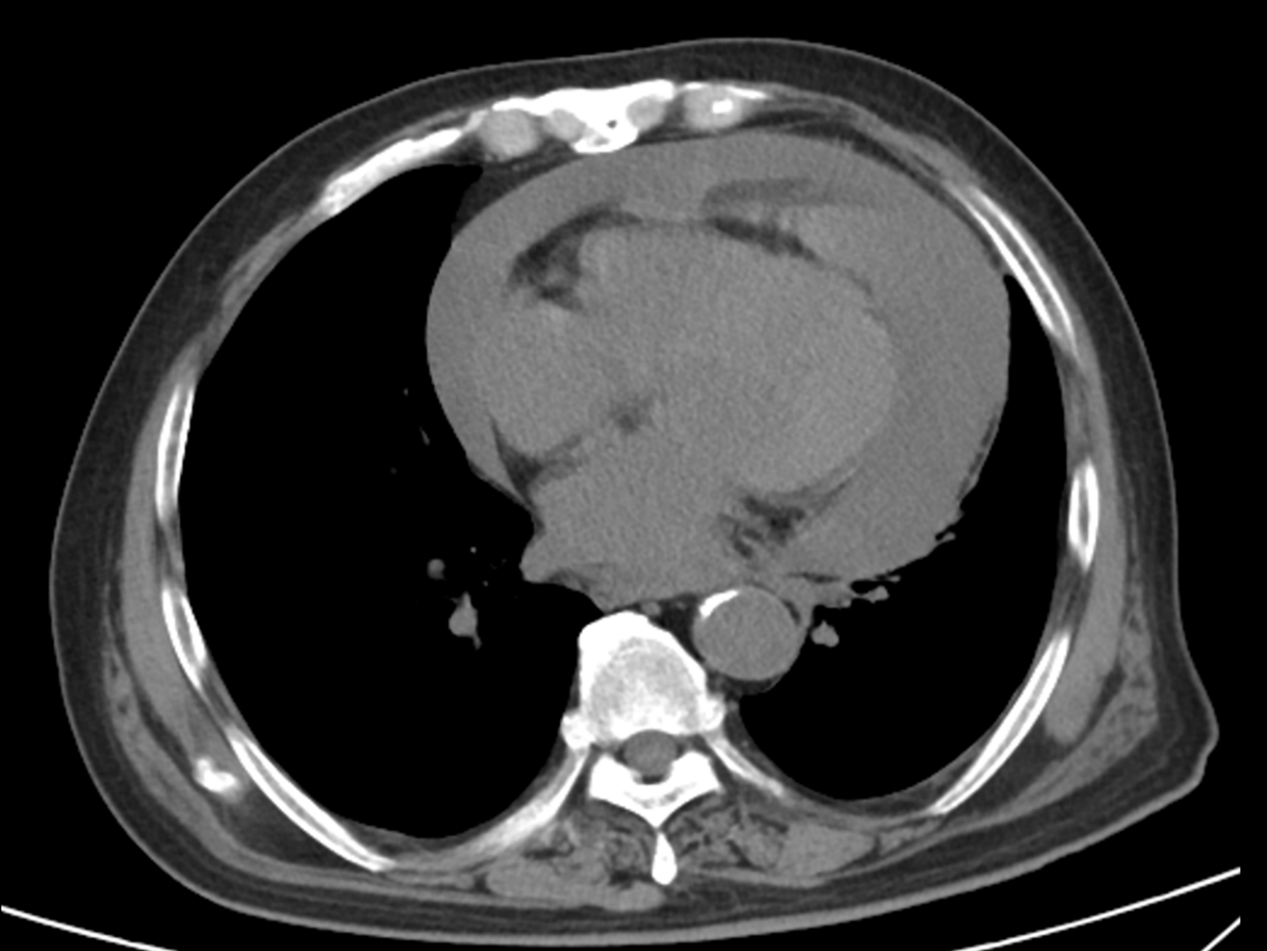
A plain CT scan of the chest (mediastinal window) on admission indicated a large pericardial effusion.

One week before admission, without any evident precipitating factors, he developed a fever and shortness of breath, with his body temperature reaching a peak of 39.8°C. This acute onset of symptoms led to his being brought to the emergency room on January 26, 2023. Upon arrival at the emergency room, his vital signs were measured: body temperature 38°C, blood pressure 87/55 mmHg, and heart rate 110 beats/min. Physical examination revealed no conspicuous dry or wet rales in both lungs, yet distant heart sounds were noted. In response to these findings, immediate actions were taken. He was placed on oxygen to address potential hypoxia, cardiac monitoring was initiated to continuously monitor his heart condition, and relevant tests and imaging examinations were promptly arranged. The subsequent chest CT scan was a pivotal diagnostic step. It disclosed two significant findings: 1. A substantial amount of pericardial effusion (**Figure 1**), which was likely contributing to his shortness of breath and other symptoms; 2. There are solid nodules in the apical-posterior segment of the left upper lobe and the posterior segment of the right upper lobe, both approximately 3 mm in size. (The patient’s tumor marker shows that the squamous cell carcinoma antigen (SCC) level is 4.9 ug/L (the normal range is 0-1.5 ug/L). Initially, the pulmonary nodules were preliminarily judged to require close short-term follow-up. Subsequently, the patient underwent a plain chest CT scan for reexamination three months later, and no significant change in the pulmonary nodules was observed compared with the previous examination result.)Given the extensive pericardial effusion, an emergency pericardial effusion puncture was performed, and the puncture fluid was bloody.

During this period, a series of laboratory tests were carried out to further evaluate his condition. Blood gas analysis results were as follows: Partial Pressure of Oxygen (PO₂):97.7 (normal range 83 - 103), Oxygenation Index (OI): 290, Partial Pressure of Carbon Dioxide (PCO₂):32.8 (normal range 35 - 45), Base Excess (BE) - 5.2 (normal range - 3 - 3), Plasma Glucose (PG) 16.8 (normal range 3.89 - 5.83), Lactic Acid (LAC) 1.6 (normal range 0.5 - 1.6).  Additionally, abnormal laboratory values included leukocytes(WBC) at 17.33×10⁹/L, neutrophil percentage(Neut%) at 79.50%, C-reactive protein(CRP) at 161.95 mg/L, heparin-binding protein(HBP) at 74.74 ng/mL (<11.4), calcitonin proteins (PCT)at 5.53 ng/mL, Interleukin-6 (IL-6) at 140.3 pg/mL, N-Terminal Pro-Brain Natriuretic Peptide (NTpro-BNP) at 29521 ng/L (normal range 0 - 300), Brain Natriuretic Peptide (BNP) at 961 ng/L (normal range 0 - 100), soluble Growth - Stimulating Expressed Gene Protein 2 at 91.07 ng/mL (normal range <35), D - dimer assay in coagulation at 15.29 μg/mL (normal range 0 - 0.55), creatinine at 1045.40 μmol/L(58 - 110), albumin at 33.50 g/L(normal range 35 - 50), creatine kinase at 47.00 U/L( normal range 55 - 175), creatine kinase isoenzyme at 2.4 U/L( normal range 0.5 - 4), and troponin I at 0.268 ng/ml( normal range 0 - 0.03), while potassium was within the normal range at 4.06 mmol/L( normal range 3.6 - 5.0). Based on these comprehensive results, he was then admitted to the intensive care unit for more in-depth treatment. Tracing back the patient's past medical history, he was hospitalized five years ago (August 30, 2018) due to type 2 diabetic nephropathy stage V (CKD stage 5) and acute left heart failure. Cardiac ultrasound at that time showed a large left ventricle, a large right atrium, weakened overall contractile activity of the left ventricular wall, and left heart function hypoplasia (LVEF 28%). Since then, he had been undergoing long-term hemodialysis treatment, and his condition had been relatively stable in the hospital. The last chest CT examination, conducted one year ago, showed emphysema, coronary sclerosis, and a small amount of pericardial effusion. Moreover, he had a history of hypertension, diabetes mellitus, and hepatitis B, but he denied a history of blood transfusion and drug allergy.

After being admitted to the ICU, a treatment plan was devised. The patient continued to receive pericardial effusion puncture and drainage to relieve the pressure caused by the effusion. Anti-infection treatment with cefoperazone-sulbactam was initiated, given the suspicion of infection, and maintenance hemodialysis was continued to manage his renal condition. The routine urine test results indicated the following: urinary white blood cells were at 3+, glucose was at 3+, and protein level was at 2+. Meanwhile, the abdominal ultrasound examination revealed no obvious signs of infection. However, routine pericardial effusion tests showed 56% neutrophils (<6%), strongly indicating infection. In light of this, the medical team decided to consult with the patient’s family about performing a mNGS test on the pericardial effusion. Simultaneously, relevant smears and cultures of the pericardial effusion were sent for examination. After admission, the patient still had a fever, and the infection index remained high. Significantly, on the same day, the mNGS results of the patient’s pericardial fluid showed *Prevotella intermedia*. As a result, the antibiotic was adjusted to cefoperazone-sulbactam combined with ornidazole for more targeted anti-infection treatment. Subsequently, the patient’s body temperature gradually returned to normal day by day, and the infection index improved compared to before (**Table 1**). Notably, the results of urine culture, sputum culture, rheumatologic system examination, and in-hospital pericardial fluid culture were all negative.

**Table 1:** Changes of various indices after treatment.

Date/Items	WBC	Neut%	CRP	PCT	Cr	BNP
2023/1/27	17.33	79.5	161.9	5.53	1045	961
2023/1/29	13.65	78.8	164.6	4.73	614	978
2023/2/7	8.77	65.8	21.2	1.09	637	954

The patient was admitted with an abnormally high D-dimer test result of 15.29 (normal range 0 - 0.55), combined with hypotension. Considering the potential risk of vascular disease, computed tomographic angiography (CTA) of large arterial vessels was performed. The CTA of large thoracic vessels revealed right middle and right lower pulmonary embolism, and poor visualization of the distal branches of the anterior endobasal segment of the left lower pulmonary artery. In response, the patient was treated with both antibiotic and anticoagulation simultaneously. After 14 days of treatment, as the infection index improved, renal function stabilized, and no obvious pericardial effusion was detected on cardiac ultrasound, the patient was discharged. Three months later, cardiac ultrasound still showed no pericardial effusion. Ultimately, the patient was diagnosed with pericardial tamponade, pericardial effusion (*Prevotella intermedia* infection), pulmonary embolism, type 2 diabetic nephropathy (CKD stage 5), heart failure, cardiac function class III (NYHA classification), type 2 diabetes mellitus, and hypertension.

## 5 Discussion

At present, the principal anaerobes isolated from patients with pericarditis predominantly consist of gram-negative bacilli, among which Bacteroides fragilis spp. stands out, accompanied by other species within the Bacteroides genus and Clostridium. Rare instances have been documented in the medical literature, such as pericardial effusion stemming from oral infections triggered by group F streptococci and hemolytic streptococci, pericardial tamponade in HIV-positive patients due to non-typhoidal Salmonella infections, and pericardial effusion associated with brucellosis and Epstein-Barr virus (EBV) infections (Carol and Steven, 2009; Paula et al., 2021; Bhaskar et al., 2022; Jacobs et al., 2022; Yubo et al., 2022; Abdelsalam et al., 2023). In Canada, an emergent diagnosis of gas-containing pericardial effusions has been made, with Prevotella, Bacteroides, Streptococcus milleri, and Streptococcus viridans being identified as the organisms that grow. Both bacillus-like bacteria and anaerobic streptococci are responsible for the generation of these gas-containing infections (Carol and Steven, 2009). Occasionally, two cases of pericarditis caused by *Prevotella intermedia* have been reported in the United States (Brook, 2009; Anna et al., 2021). Remarkably, one of them was initially misdiagnosed as tuberculous pericardial effusion. *Prevotella intermedia*, a species belonging to the Prevotella genus, currently encompasses 43 species and 1 subspecies. It has been strongly correlated with periodontitis and acute necrotizing ulcerative gingivitis (Xi et al., 2025), and may potentially play a crucial role in oral mucosal carcinogenesis (Liu et al., 2025). Moreover, there have been reports of purulent pleural effusions resulting from *Prevotella intermedia* infections (Psit et al., 2025). The typical treatment modalities for *Prevotella intermedia* infection commonly involve nitroimidazoles, penicillins, or quinolones.

In the current case under discussion, the patient had a history of heart failure and renal failure. The onset of the disease was manifested by fever and shortness of breath. Laboratory investigations disclosed a significant elevation in inflammatory biomarkers, and a chest CT scan revealed a copious amount of pericardial effusion. Given the dearth of compelling evidence suggesting infection in other bodily regions, the probability of an infectious etiology was judged to be higher than that attributed to heart failure and renal failure. Consequently, infected pericardial effusion was regarded as the primary diagnosis. Following the adjustment of the anti-infection treatment regimen based on the mNGS results, the patient’s clinical symptoms and infection indices improved markedly, and the patient was ultimately discharged in a stable condition. The origin of the *Prevotella intermedia* infection in this particular patient remains elusive. There have been prior cases of rapidly progressing pericardial tamponade due to oropharyngeal polymicrobial infections in the absence of a readily identifiable primary source of infection (Mukul et al., 2014). The patient in this case had a long-standing subclavian hemodialysis catheter inserted. However, there were no conspicuous signs of local infection, and the patient continued hemodialysis using this catheter for one year without subsequent reports of infection in other patients. Tracing back the patient’s history of disease onset, a history of dental caries and diabetes was uncovered. Thus, it cannot be discounted that oral infection might have led to the pericardial effusion caused by *Prevotella intermedia* infection.

In this case, the patient was prone to misdiagnosis as having non-infectious pericardial effusion due to heart failure and renal failure. The patient underwent urgent pericardial effusion puncture and drainage in the emergency department, and the diagnosis was finally clarified based on a comprehensive review of the medical history and supplementary examinations. Notably, mNGS played a pivotal role in swiftly identifying the pathogen in the clinical setting. Currently, mNGS is of immense significance in enhancing the diagnosis and treatment of infectious diseases. A meta-analysis has demonstrated that, in comparison to the culture method, mNGS exhibits a higher detection rate in pus and cerebrospinal fluid (CSF) samples, and considerably more anaerobes were detected in CSF samples (Xuyang et al., 2024).

In conclusion, while previous medical literature has recorded cases of pericardial effusion associated with Prevotella species, it is crucial to emphasize that the current study presents the first-ever report in China of pericardial tamponade and effusion directly caused by *Prevotella intermedia*. This finding substantially augments our understanding of infectious pericardial disorders and broadens the spectrum of potential causative agents that clinicians need to take into account. The identification of *Prevotella intermedia* as the underlying cause in this case was a challenging endeavor. This pathogen, being an opportunistic one, thrives in anaerobic settings and has often eluded conventional diagnostic means. Routine laboratory tests, while forming the foundation of medical diagnostics, have sometimes struggled to detect their presence, thereby adding complexity to the identification process. However, with the advent and application of advanced second-generation sequencing (mNGS) technology, the pathogen was successfully revealed. This not only showcases the power of mNGS but also highlights the importance of having a diverse toolkit in modern medicine to address complex diagnostic scenarios. Nevertheless, the implications and challenges stemming from this discovery are extensive. Firstly, due to the rarity of this particular diagnosis, many clinicians may not have had prior exposure or in-depth knowledge, which could impede their ability to swiftly recognize and diagnose *Prevotella intermedia* infections. Such delays or misinterpretations could have serious ramifications for patients. Secondly, the patient in question had a multifaceted medical history, encompassing heart failure, renal failure, diabetes, and hypertension. These pre-existing conditions not only heightened his vulnerability to opportunistic infections but also muddled the clinical picture, making it arduous to differentiate infection symptoms from those of the underlying ailments. A collaborative, multidisciplinary approach involving specialists from cardiology, nephrology, endocrinology, and infectious diseases is, therefore, essential for accurate diagnosis and optimal treatment. Finally, prevention emerges as a cardinal aspect in the fight against opportunistic infections. Considering the possible origins of *Prevotella intermedia*, like the oral cavity and hemodialysis catheters, implementing targeted preventive strategies is of utmost importance. For patients with underlying diseases, promoting oral hygiene awareness and ensuring regular dental check-ups can appreciably curtail the risk of oral pathogen spread. Likewise, maintaining strict aseptic protocols during hemodialysis and vigilantly monitoring for any signs of catheter-related infections are indispensable. In summary, the discovery of this initial case of *Prevotella intermedia*-induced pericardial effusion marks a notable milestone, yet it also sounds an alarm for the medical community. It is incumbent upon researchers, clinicians, and healthcare administrators to join forces and work in tandem to surmount the hurdles presented by this uncommon but potentially life-threatening infection. Only through such concerted efforts can we anticipate enhancing the prognosis and quality of life for patients at risk.

## Data Availability

The datasets presented in this study can be found in online repositories. The names of the repository/repositories and accession number(s) can be found in the article/supplementary material.
